# A process-guided uncertainty-aware deep learning framework for reliable and interpretable industrial fault diagnosis

**DOI:** 10.1371/journal.pone.0349385

**Published:** 2026-06-02

**Authors:** Babar Hayat, Shabeer Ahmad, Muhammad Asfandyar Shahid, Adil Khan, Md. Rajibul Islam, Md Shohel Sayeed, Yasir Ullah

**Affiliations:** 1 School of Information Engineering, Xi’an Eurasia University, Xi’an, Shaanxi, China; 2 School of Electronic Engineering, Beijing University of Posts and Telecommunications, Beijing, China; 3 School of Automation and Electrical Engineering, University of Science and Technology Beijing, Beijing, China; 4 Department of Computer Science and Engineering, Bangladesh University of Business and Technology, Dhaka, Bangladesh; 5 Centre for Intelligent Cloud Computing, CoE for Advanced Cloud, Faculty of Information Science and Technology, Multimedia University, Bukit Beruang, Melaka, Malaysia; 6 Centre for Wireless Technology, Faculty of AI and Engineering, Multimedia University, Cyberjaya, Selangor, Malaysia; Gachon University, KOREA, REPUBLIC OF

## Abstract

Timely fault detection is essential for safety, product quality, and energy efficiency in advanced industrial processes. However, many existing fault diagnosis methods insufficiently exploit process structure and sensor reliability, which limits their robustness and practical usefulness for process engineers. This study presents an improved framework SAU-PGA-CNN-BiLSTM that first couples Convolutional Neural Networks and Bidirectional Long Short-Term Memory layers to extract multivariate temporal dynamics and spatial correlations of the process data, secondly a process guided and sensor-aware attention mechanism is introduced which embeds process centrality, sequence level sensor reliability and uncertainty to the attention learning, to suppress unreliable channels and bias towards informative and stable sensors. In addition, Monte Carlo dropout with sensor prior-conditioning is used to provide calibrated confidence estimates that reflect both predictive uncertainty and sensor reliability. Finally, two lightweight sigmoid output heads perform fault detection and diagnosis combinedly, promoting mutual reinforcement between the tasks. Validated on the Tennessee Eastman Process benchmark, the proposed framework outperforms baselines model and achieves 93.6% multiclass diagnosis accuracy with 94.0% F1 score. After temperature scaling, the proposed model also demonstrates improved calibration compared with an otherwise identical model without sensor awareness, reducing negative log-likelihood from 0.197 to 0.182, Brier score from 0.101 to 0.095, and expected calibration error from 0.040 to 0.037. Attention visualizations further show that the model focuses on process-relevant and reliable sensors, supporting reliable industrial fault diagnosis.

## 1. Introduction

Detecting and diagnosing abnormal events quickly in large industrial processes is crucial for safety, maintaining product quality, and improving energy efficiency [1]. As modern industrial processes use more and more dense sensor networks, they generate and store huge amounts of process data every day, creating a unique chance for intelligent monitoring [2]. However, the complex, nonlinear, and constantly changing nature of industrial systems makes fault detection and diagnosis (FDD) quite challenging [3]. Faults that are missed or diagnosed may propagate at a very fast rate, resulting in damaged equipment, environmental risks, and significant financial loss [4]. The common process monitoring methods which have been used extensively in fault detection and diagnosis (FDD) of industrial systems include Principal Component Analysis (PCA) Partial least squares (PLS) and Multivariate Statistical Process Control (MSPC) [5–7]. Data-driven techniques, including multivariate statistical process monitoring and machine learning methods, have therefore gained substantial attention due to their flexibility and reduced dependence on explicit process models. However, classical methods such as PCA, PLS, and shallow classifiers typically assume linear relationships and lack the capacity to capture complex temporal dependencies and fault propagation patterns [8,9]. Moreover, such procedures do not provide a practical understanding of the underlying causes of failures, lowering the usefulness of the operator intervention and troubleshooting [3]. The recent surging popularity of deep learning technologies has spawned advanced data-intensive models of monitoring and diagnosis of industrial processes. CNNs are also skilled at describing local spatial correlations of sensors, whereas Recurrent Neural Networks (RNNs), especially LSTM units are skilled at describing temporal dependencies [10,11]. Hybrid models that combine CNNs with LSTMs or BiLSTMs have shown notable improvements in accuracy on benchmark datasets like the Tennessee Eastman Process (TEP). Nevertheless, more powerful deep learning models are also costly, in the cost of training and running them, which makes them impractical to apply in industrial contexts with highly constrained time, latency, and hardware requirements [12,13].

More importantly, the vast majority of deep neural networks remain black boxes, which do not provide much interpretability to process engineers [14]. This transparency deficiency makes automated monitoring system implementation challenging as operators require beyond alerts, they want to know what variables and time periods are behind the observed anomalies [15]. Attention mechanism has been proposed as a promising technique that can provide interpretability, i.e., make the internal workings of neural networks transparent by giving priority ratings to input elements and time steps [16]. However, process monitoring models that use attention are often based on multi-head attention or transformer designs, which add complexity and computational cost to the models [17]. Beyond predictive accuracy and interpretability, practical industrial fault detection and diagnosis (FDD) systems must also be able to quantify and communicate prediction confidence [18]. In dynamic and noise-dominated industrial environments, knowing whether a model is uncertain about an alarm is critical, as this directly influences risk-aware operational decisions and operator intervention strategies [19]. Although uncertainty quantification techniques such as Monte Carlo dropout enable deep learning models to provide calibrated confidence estimates alongside their predictions [20,21], such capabilities remain largely underexplored in current data-driven process monitoring and diagnosis literature. Consequently, there is a growing need for FDD frameworks that jointly deliver accurate fault predictions, reliable uncertainty-aware outputs, and interpretable diagnostic explanations, while remaining computationally efficient for real-time industrial deployment.

To overcome these issues, we propose sensor-aware uncertainty and process-guided attention mechanism CNN-BiLSTM (SAU-PGA-CNN-BiLSTM) framework is specifically intended for accurate and interpretable fault detection and diagnosis in complicated industrial situations. A process-guided attention mechanism dynamically highlights fault-relevant sensor–time patterns, producing interpretable attention heatmaps that are consistent with known process behavior and facilitate faster root-cause analysis. Furthermore, the combination of MC dropout–based uncertainty estimation and post-hoc temperature scaling yields well-calibrated confidence scores, enabling confidence-aware alarm handling and selective decision deferral in safety-critical settings.

## 2. Related work

Traditional methods for process monitoring and fault detection are based on statistical process control and latent variable models. Principal Component Analysis (PCA) and its dynamic variants [22] have been widely used to extract complex sensor data, making it easier to spot when a process is behaving abnormally. The Squared Prediction Error (SPE) and Hoteling’s *T*^2^ are commonly used for process monitoring [23], but since they rely on linear assumptions, they struggle with processes that behave in nonlinear ways. The Kernel PCA [24] and the Independent Component Analysis (ICA) [25] has some advantages, but in many cases, they require more computing power and more careful parameter adjustment. Multivariate statistical process control (MSPC) methods like the Partial Least Squares (PLS) and Canonical Correlation Analysis (CCA) provides more flexibility as they are more flexible in modelling the relationships between inputs and outputs [7,26]. However, they continue to have difficulties in offering time-based details or in detecting causes of roots especially in complicated or non-linear systems. Machine learning approaches based on data have been investigated to support FDD [27,28], including support vector machines (SVMs) random forests and gradient boosting, yet such approaches are demanding of manually designed features, and they do not account for the important temporal relationships that are necessary to analyze fault progression.

Deep learning has greatly improved fault detection and diagnosis (FDD) in recent years. Convolutional Neural Networks (CNNs) have shown great ability to capture spatial relationships across sensor networks [29]. By applying convolutional filters to input data, CNNs can learn local fault patterns that global linear method often misses. However, CNNs by themselves struggle to handle time-based dependencies. Recurrent Neural Networks (RNNs), especially LSTM networks, are often used to model temporal dynamics in process data [30]. LSTMs help solve the vanishing gradient problem found in basic RNNs and work well for tracking trends and detecting anomalies over long sequences [31]. Combining CNNs and LSTMs into hybrid models takes advantage of both types, capturing spatial and time-related patterns at the same time [32]. Furthermore, the study found that a hybrid model improved fault detection and diagnosis on the TEP dataset compared to using either model alone [33]. Bidirectional LSTMs (BiLSTM) improve temporal modeling by processing input sequences both forward and backward, which helps in detecting subtle or long-range fault patterns [34]. Despite these improvements, most existing CNN–RNN-based methods remain purely data-driven, offering limited interpretability and providing only point predictions without explicit measures of predictive confidence.

Attention mechanisms have become popular for making deep neural networks easier to understand in areas like natural language processing and time-series analysis [35]. In industrial fault detection and diagnosis (FDD), methods like additive attention and scaled-dot product attention are used to identify the most important variables and time points [36]. By giving dynamic weights to sensor and time pairs, attention layers help reveal the cause-and-effect relationships behind process faults. Recent studies have combined attention mechanisms with CNN–LSTM architectures to boost both accuracy and interpretability [37]. However, many of these models rely on multi-head or transformer-based attention [38], which greatly increases complexity and resource use. Moreover, existing attention-based FDD methods generally learn attention weights solely from data, without incorporating process knowledge or sensor reliability information. As a result, attention maps may overemphasize noisy or unstable sensors, reducing robustness and potentially misleading operators during fault analysis. Beyond accuracy and interpretability, reliable industrial deployment requires models to express prediction uncertainty, especially in safety-critical environments. Uncertainty-aware learning has been studied in broader machine learning contexts using Bayesian neural networks, ensemble methods, and Monte Carlo (MC) dropout [39], In industrial FDD, uncertainty estimates can support risk-aware alarm handling, selective prediction, and operator decision-making. Most deep learning models for fault detection and diagnosis (FDD) only give point estimates and don’t show how confident or reliable their predictions are. In high-risk industrial environments [40], it is important to tell the difference between alarms that are highly confident and those that are less certain, to manage risks better and use resources more efficiently. Monte Carlo dropout [41] has become a practical way to approximate Bayesian inference in neural networks. By keeping dropout active during inference and averaging several random forward passes, the model can estimate both the average prediction and its uncertainty. Furthermore, post-hoc calibration techniques such as temperature scaling, which are effective in improving probabilistic reliability [42], are rarely integrated into industrial FDD pipelines. Overall prior research has achieved notable progress in fault detection and diagnosis using deep learning and attention mechanisms. However, there are still critical gaps limited integration of process knowledge into attention mechanisms, lack of sensor-aware uncertainty handling, and insufficient focus on probabilistic calibration and risk-aware decision support. To bridge these gaps, this paper proposes a Sensor-Aware Uncertainty and Process-Guided Attention CNN–BiLSTM (SAU-PGA-CNN-BiLSTM) framework that jointly addresses accuracy, interpretability, and reliability. By embedding process topology and sensor reliability into the attention mechanism and explicitly modeling predictive uncertainty through MC dropout and temperature scaling, the proposed approach advances the state of the art toward deployable, trustworthy industrial FDD systems. Our contributions are summarized as follows:

- A unified SAU-PGA-CNN-BiLSTM framework is proposed that jointly performs multiclass FDD while explicitly quantifying predictive uncertainty through MC dropout. Unlike conventional pipelines that treat diagnosis and uncertainty estimation as separate stages, the proposed approach delivers fault predictions and calibrated confidence estimates within a single end-to-end inference process.
- A novel attention design is introduced that integrates sensor-aware uncertainty weighting with process-guided priors, allowing the model to dynamically emphasize reliable and process-critical sensors while down-weighting noisy or weakly informative channels. This mechanism embeds domain knowledge directly into the learning process, yielding attention patterns that are physically meaningful and consistent with known fault propagation behavior.
- The proposed framework combines convolutional layers to capture inter-sensor correlations and local temporal patterns with stacked BiLSTM layers to model long-range fault evolution. This architectural synergy enables accurate diagnosis of complex, distributed faults while maintaining moderate computational complexity suitable for online industrial monitoring.
- Extensive experiments on the Tennessee Eastman Process dataset demonstrate that the proposed framework achieves high fault detection and diagnosis accuracy, improved calibration and uncertainty behavior, and clear, interpretable attention visualizations. The model operates faster than typical industrial sampling intervals, supporting its practical applicability in real-time, safety-critical industrial environments.

## 3. Proposed network design

The proposed SAU-PGA-CNN-BiLSTM network integrates four well-studied deep-learning blocks, each tailored to a specific function in real-time industrial fault analysis.

### 3.1. Two-dimensional convolutional neural network

One-dimensional CNNs use convolutional filters to slide across sequential data, capturing local feature interactions between sensor readings [43]. In this study, CNN layers function as spatial feature extractors, automatically detecting spatial correlated sensor behaviors that indicate possible fault conditions. Given an input vector of sensor measurements at time step *t* can written as:


$$\begin{array}{c} x_{t}={\left[x_{t}^{\left(1\right)},x_{t}^{\left(2\right)},\ldots ,x_{t}^{\left(F\right)}\right]}^{T}\in {\mathbb{R}}^{F} \end{array}$$ (1)

Each convolutional kernel $K_{c}\in {\mathbb{R}}^{F\times d}$ calculates a feature map as follows:


$$\begin{array}{c} z_{t,c}=\sigma \left(\sum_{f=1}^{F}\sum_{k=1}^{d}K_{c}(f,k)x_{t}^{\left(f\right)}+b_{c}\right),\;c=1,\ldots ,C \end{array}$$ (2)

Where σ(·) denotes the ReLU activation function, and two convolution layers are stacked to produce the spatially encoded features. The tensor can be written as:


$$\begin{array}{c} Z\in R^{T\times C} \end{array}$$ (3)

### 3.2. Bidirectional long-short term memory networks

Bidirectional LSTM (BiLSTM) networks handle sequences in both forward and reverse temporal directions, incorporating rich context into feature representations [44]. The BiLSTM layers serve as a temporal encoder, successfully capturing complicated temporal dependencies and discriminating between transient fluctuations and permanent fault deviations. For each direction (→,←), the LSTM hidden state and cell state at time *t* are updated as follows:


$$\begin{array}{c} f_{t}=\sigma \left(W_{f}z_{t}+U_{f}h_{t-1}+b_{f}\right) \end{array}$$ (4)

$$\begin{array}{c} i_{t}=\sigma \left(W_{i}z_{t}+U_{i}h_{t-1}+b_{i}\right) \end{array}$$ (5)

$$\begin{array}{c} {\tilde{c}}_{t}=\tanh\left(W_{c}z_{t}+U_{c}h_{t-1}+b_{c}\right) \end{array}$$ (6)

$$\begin{array}{c} c_{t}=f_{t}⊙c_{t-1}+i_{t}⊙{\tilde{c}}_{t} \end{array}$$ (7)

$$\begin{array}{c} o_{t}=\sigma \left(W_{o}z_{t}+U_{o}h_{t-1}+b_{o}\right) \end{array}$$ (8)

$$\begin{array}{c} h_{t}=o_{t}⊙\tanh\left(c_{t}\right) \end{array}$$ (9)

where ⊙ denotes element-wise multiplication. The forward and backward hidden states are then combined as:


$$\begin{array}{c} h_{t}=\left[{\vec{h}}_{t}∥{\overset{\leftarrow }{h}}_{t}\right]\in {\mathbb{R}}^{2H} \end{array}$$ (10)

### 3.3. Attention mechanism

The additive attention method adds dynamic relevance weights to different time steps, allowing the model to select the most relevant chunks of the input sequence [45]. Attention serves as the interpretability module, giving transparent representations of the decision-making process using attention-weighted saliency maps to aid operator comprehension. Attention alignment scores and weights are calculated as:


$$\begin{array}{c} e_{t}=v^{⊤}\tanh\left(W_{a}h_{t}+b_{a}\right),\;\alpha_{t}=\frac{\exp(e_{t})}{\sum_{k=1}^{T}\exp(e_{k})},\;\sum_{t=1}^{T}\alpha_{t}=1 \end{array}$$ (11)

The aggregated context vector is obtained by:


$$\begin{array}{c} c=\sum_{t=1}^{T}\alpha_{t}h_{t}\in {\mathbb{R}}^{2H} \end{array}$$ (12)

### 3.4. Monte Carlo dropout for uncertainty estimation

Monte Carlo dropout is a useful technique for assessing predictive uncertainty. It involves conducting numerous stochastic forward passes with active dropout layers during inference [46]. It provides the model with a confidence estimation mechanism, distinguishing between high-confidence predictions suitable for automated action and low-confidence predictions. With dropout rate *p*_*drop*_, predictions from *N*_*MC*_ stochastic forward passes yield a set of probabilities $\{p^{\left(i\right)}{\}}_{i=1}^{N_{MC}}$. The predictive mean and variance are:


$$\begin{array}{c} \hat{p}=\frac{1}{N_{MC}}\sum_{i=1}^{N_{MC}}p^{\left(i\right)},\;{\hat{\sigma }}^{2}=\frac{1}{N_{MC}}\sum_{i=1}^{N_{MC}}(p^{\left(i\right)}-\hat{p}{)}^{2} \end{array}$$ (13)

A high predictive variance ${\hat{\sigma }}^{2}$ indicates low confidence, enabling a risk-aware decision-making strategy. This combination results in a robust, interpretable, and uncertainty-aware system specifically intended for effective real-time fault detection and diagnosis in a complex industrial environment.

## 4. Methodology

### 4.1. Proposed framework overview

**Fig 1:**
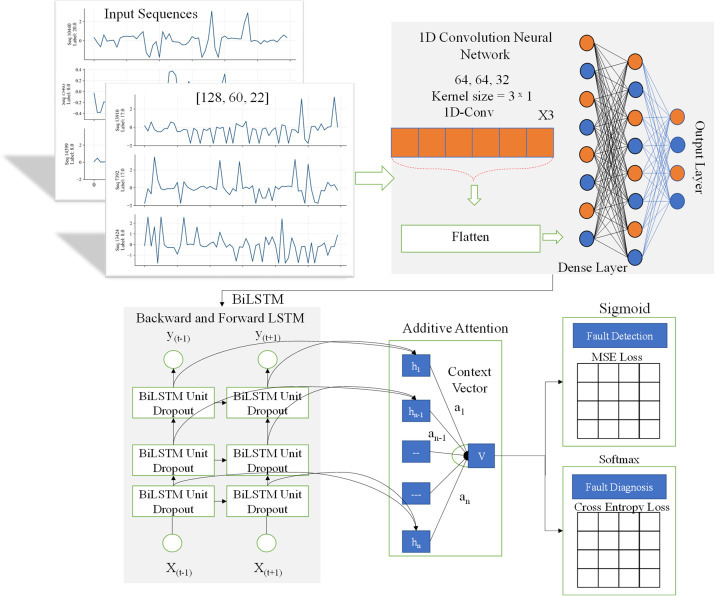
Proposed SAU-PGA-CNN-BiLSTM framework for industrial fault detection and diagnosis.

This study proposed a hybrid deep learning framework called SAU-PGA-CNN-BiLSTM aimed for robust fault detection and diagnosis in complex industrial processes. The framework is designed to jointly address key challenges in industrial processes modeling of complex multivariant temporal dynamics and spatial correlations of the process data, accounting for heterogenous sensor reliability, and providing calibrated confidence estimates alongside diagnostic decisions. The model integrates convolutional layers and bidirectional LSTM layers to extract spatial correlations and understand time-related patterns of the data, and a process guided sensor-aware attention mechanism within a unified framework. Using time-series industrial sensors data, the framework performs fault detection and multiclass diagnosis, while simultaneously estimating predictive uncertainty through Monte Carlo dropout and post-hoc calibration. An overview of the proposed methodology is shown Fig 1.

### 4.2. Framework architecture

The proposed SAU-PGA-CNN-BiLSTM framework processes multivariate sensor time-series data $𝐗\in R^{T\times F}$, where *T* is the sequence length and *F* is the features at each time step. It uses a hierarchical approach that combinedly captures both spatial and temporal relationships, while providing interpretable and uncertainty-aware predictions. First, one-dimensional convolutional layers extract spatial feature maps $𝐙\in R^{C\times T}$ which summarize local interactions among variables. These features are then reshaped to ${𝐙}^{⊤}\in {\mathbb{R}}^{T\times C}$ to serve as input to the subsequent temporal modeling stage. To capture long-range temporal dependencies and fault propagation behavior, the reshaped features are passed into a bidirectional long short-term memory (BiLSTM) network. The BiLSTM processes the sequence in both forward and backward directions, enabling the model to learn temporal dependencies related to fault onset and evolution. For each time step t, the hidden state representation is given by:


$$\begin{array}{c} h_{t}=\left[{\vec{h}}_{t};{\overset{\leftarrow }{h}}_{t}\right] \end{array}$$ (14)

Where ${\vec{h}}_{t}\;{\overset{\leftarrow }{h}}_{t}$, shows the forward and backward hidden states respectively. An additive attention mechanism is then employed to identify the most informative time steps and sensor-derived features for fault diagnosis. Unlike conventional attention mechanisms, the attention logits in this framework are modulated by process-guided and sensor-aware priors, including process centrality, sensor reliability, and sensor uncertainty. These priors bias the attention weights toward reliable and process-consistent measurements while suppressing noisy or sensors data. The normalized attention weights $\alpha_{t}$ are computed and used to aggregate the BiLSTM outputs into a context vector, that highlight the temporal importance of each *h*_*t*_.


$$\begin{array}{c} c=\sum_{t=1}^{T}\alpha_{t}h_{t} \end{array}$$ (15)

The resulting context vector c captures the most relevant spatiotemporal features for fault detection and diagnosis in a physically interpretable manner. The context vector is shared by two output heads to enable joint learning of fault detection and fault diagnosis. A sigmoid-activated output layer performs binary fault detection can be written as:


$$\begin{array}{c} {\hat{y}}_{f}=\sigma \left(w_{f}^{⊤}c+b_{f}\right) \end{array}$$ (16)

And SoftMax layer for multi-class fault diagnosis


$$\begin{array}{c} {\hat{y}}_{d}=\text{softmax}\left(W_{d}c+b_{d}\right) \end{array}$$ (17)

This shared-representation strategy improves learning efficiency and ensures consistency between detection and diagnosis tasks. To enhance reliability in safety-critical industrial settings, MC dropout is employed during inference to approximate Bayesian uncertainty. Multiple stochastic forward passes generate predictive distributions rather than point estimates, allowing computation of predictive means and variances for both detection and diagnosis outputs. This uncertainty information provides calibrated confidence measures that support risk-aware decision-making and operator intervention. Intuitively, sensor-aware uncertainty reflects both the model predictive confidence and the reliability of the underlying sensor signals. Predictions supported by stable and consistent sensors result in lower uncertainty, whereas noisy or unreliable sensor inputs lead to higher uncertainty. This allows operators to distinguish between confident and potentially unreliable decisions, thereby supporting safer and more informed process monitoring. To further improve probability calibration, post-hoc temperature scaling is applied following standard practice. The temperature parameter is fitted exclusively on the validation split, which is strictly separated from both training and test data to avoid information leakage. A single global temperature is learned for the multiclass fault diagnosis output and shared across all fault classes. At test time, predictive uncertainty is first estimated by averaging logits across MC-dropout forward passes, after which temperature scaling is applied only to the aggregated logits before computing calibrated SoftMax probabilities. In summary, the proposed SAU-PGA-CNN-BiLSTM framework integrates spatiotemporal feature extraction, process-guided sensor-aware attention, and uncertainty-aware calibration within a unified architecture. The CNN and BiLSTM layers capture spatial correlations and temporal dynamics, while the process-guided attention mechanism incorporates process centrality, sensor reliability, and uncertainty priors to emphasize informative and stable sensor signals. The model jointly performs fault detection and diagnosis using shared representations, ensuring consistency and efficiency. Furthermore, Monte Carlo dropout combined with temperature scaling provides calibrated confidence estimates for risk-aware decision-making. This integrated design enables accurate and reliable fault diagnosis suitable for real-time industrial applications.

## 5. Description of TEP data

**Fig 2:**
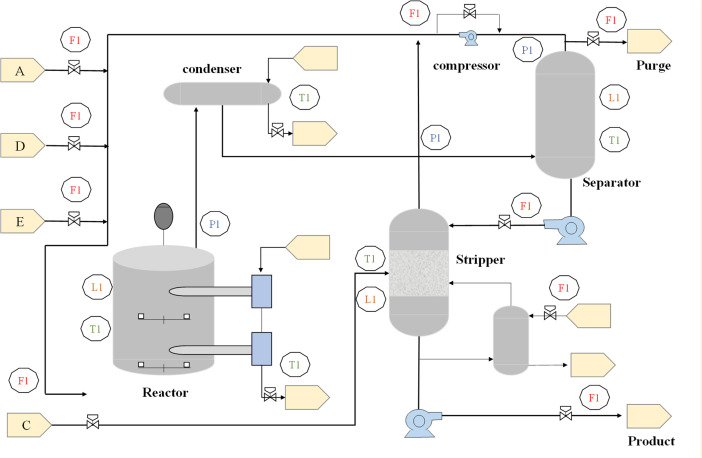
Schematic diagram of Tennessee Eastman Process (TEP).

Vogel originally provided the Tennessee Eastman process (TEP) dataset, and Down is the chemical process simulation model [47], which is commonly used as a benchmark in the field of process control to compare various problem detection and diagnosis methodologies [33]. TEP is made up of five functioning units: a reactor, condenser, compressor, separator, and stripper, as shown in Fig 2. The process model represents indiscriminate relationships in operational units, and generates two sets of training and test data, comprising normal and faulty data are generated from the TEP process.

Let *x*(*t*) denote the process data, with 52 features for each time series sample containing 41 measured and 11 modified variables. These 52 variables were collected at 3-minute intervals during the running process. There are 25 hours of training data and 48 hours of testing data, with each fault appearing after one hour of training and eight hours of testing. There is one class for fault-free conditions and twenty classes for faulty states as shown in Table 1.

**Table 1:** Description of fault types in TEP.

Fault Names	Details	Fault Types
IDV 1	A/C feed ratio, B component Constant	Step
IDV 2	B composition, A/C ratio constant	Step
IDV 3	D feed Temperature	Step
IDV 4	Cooling water reactor Inlet temperature	Step
IDV 5	Condenser cooling water Inlet Temperature	Step
IDV 6	Feed loss A	Step
IDV 7	Pressure loss C	Step
IDV 8	Feed component A, B, C	Random Variation
IDV 9	Feed Temperature D	Random Variation
IDV 10	Feed Temperature C	Random Variation
IDV 11	Cooling water reactor Inlet Temperature	Random Variation
IDV 12	Cooling water condenser Inlet Temperature	Random Variation
IDV 13	Dynamic Reaction	Slow Drifting
IDV 14	Cooling water valve Reactor	Sticking
IDV 15	Cooling water valve Condenser	Sticking
IDV 16	Unknown	Unknown
IDV 17	Unknown	Unknown
IDV 18	Unknown	Unknown
IDV 19	Unknown	Unknown
IDV 20	Unknown	Unknown
IDV 21	Fixed valve at Steady-state position	Constant Position

### 5.1. Data collection and preprocessing

The data used in this study is a TEP dataset generated by a sophisticated process simulation model. Tennessee Eastman Process (TEP) data is a simulated real-life industrial data set that captures the plant activity in both normal and faulty conditions. The simulations represent different operating scenario and faults are caused randomly in the simulation. The dataset comprises of 25,101 samples and 20 failure circumstances. To have an effective fault detection and diagnosis, TEP dataset preprocessing involves a number of key steps to be followed in order to have clean and structured data to be analyzed. First, the binary labels are obtained to fault detection by encoding the fault number by a binary indicator, where 0 indicates normal conditions and 1 indicates faulty conditions. Conversely, there are multi-class labels that are developed across classifications of faults. Advanced feature engineering approaches are used, including computing time characteristics such as rolling average, standard deviation, skewness, and kurtosis to capture time-dependent patterns. To achieve uniformity in feature scaling, the data was standardized through Standard Scalar. Furthermore, the sliding window technique generates a 100-step sequence, allowing the model to learn from time dependencies. Finally, stratified sampling was used to divide the data into training, validation, and test sets, to ensure the balance representation of normal and faulty data across all sets.

### 5.2. Training phase of the proposed framework

During training, raw multivariate time-series data from the Tennessee Eastman Process (TEP) benchmark is first preprocessed. All sensor variables are normalized using z-score normalization to ensure numerical stability and consistent scaling. The dataset contains 52 process variables and several fault types. Each sample is labeled by its fault number, and for binary fault detection, samples with a fault number greater than 0 are labeled as 1 (faulty), and others as 0 (normal). To capture the sequential nature of the industrial process data, fixed-length overlapping windows of size *T* = 50 are created using a sliding window method. Each sequence $𝐗\in R^{T\times F}$, where *F* = 52 is the number of features, is paired with its corresponding fault label for classification. The sequences are randomly shuffled and split into 80% training/validation and 20% test data, with the training portion further divided into 90% training and 10% validation using a fixed random seed for reproducibility; all reported results are obtained on the held-out test set, and normalization statistics are estimated from the training data only to prevent information leakage. Model parameters are optimized using AdamW (learning rate 0.001, weight decay 10^−4^ a batch size of 64 with a warmup–cosine learning rate schedule (6 warmup epochs), trained for up to 48 epochs with early stopping (patience = 12) based on validation loss. Class imbalance is handled through class-weighted cross-entropy for diagnosis (inverse square-root frequency weighting) and a weighted random sampler that oversamples rare fault classes. To improve robustness, Gaussian input noise is added during early epochs, uncertainty-aware sensor dropout is applied with dropout probabilities proportional to sensor uncertainty priors, gradient clipping is used, and mixed-precision training is enabled. The framework is trained in a multi-task manner, jointly performing fault diagnosis and fault detection with a composite loss that combines class-weighted cross-entropy, binary detection loss, and regularization terms that penalize excessive predictive variance and discourage high-confidence predictions from relying on unreliable sensors. The model is trained using combined loss function


$$\begin{array}{c} L_{\text{Total}}=L_{\text{Detection}}+L_{\text{Diagnosis}} \end{array}$$ (18)

$$\begin{array}{c} \text{BCE}\left({\hat{y}}_{\text{Detection}},y_{\text{Detection}}\right)+\text{WCE}\left({\hat{y}}_{\text{Diag}},y_{\text{Diag}}\right) \end{array}$$ (19)

where BCE is the binary cross-entropy loss for fault detection, and WCE is the weighted cross-entropy loss for diagnosis, with class weights set inversely proportional to class frequency. Training runs for 40 epochs using the Adam optimizer with a learning rate of 0.001, a batch size of 64, and early stopping based on validation loss.

### 5.3. Evaluation phase of the proposed framework

During evaluation, two inference variants are evaluated using the same trained backbone to ensure a controlled comparison. The proposed SA setting activates the full process-guided, sensor-aware attention by incorporating the process centrality $A_{bias,}$ sensor reliability *R*_*rel*_ and uncertainty priors into the attention logits. As a baseline, a No-SA variant disables the sensor-aware terms at inference while keeping the architecture and learned parameters unchanged, thereby isolating the effect of sensor awareness on calibration and uncertainty behavior. Predictive uncertainty is estimated using Monte Carlo dropout with $T_{MC}=50$ stochastic forward passes per test sample, and the mean softmax probability is used to compute predictive entropy and related uncertainty measures. Probability calibration is further improved via post-hoc temperature scaling, with the temperature fitted on the validation set only and applied consistently to both SA and No-SA outputs at test time, for clarity, a single global temperature parameter is used across all experiments. The temperature is applied uniformly to all classes and samples during inference, ensuring consistent and stable probability calibration, with N = 20 samples, the predicted fault probability and diagnosis results are averaged:


$$\begin{array}{c} {\bar{y}}_{f}=\frac{1}{N}\sum_{i=1}^{N}{\hat{y}}_{f}^{\left(i\right)},\;{\hat{y}}_{f}^{\text{bin}}=𝕀\left({\bar{y}}_{f}>0.5\right) \end{array}$$ (20)

$$\begin{array}{c} {\bar{y}}_{d}=\frac{1}{N}\sum_{i=1}^{N}\text{softmax}\left({\hat{y}}_{d}^{\left(i\right)}\right),\;{\hat{y}}_{d}=\text{arg}\max{\bar{y}}_{d} \end{array}$$ (21)

The predictive variance is also calculated as:


$$\begin{array}{c} \text{Var}(y_{f})=\frac{1}{N}\sum_{i=1}^{N}({\hat{y}}_{f}^{\left(i\right)}-{\bar{y}}_{f}{)}^{2},\;\text{Var}(y_{d})=\frac{1}{N}\sum_{i=1}^{N}\|{\hat{y}}_{d}^{\left(i\right)}-{\bar{y}}_{d}{\|}^{2} \end{array}$$ (22)

**Fig 3:**
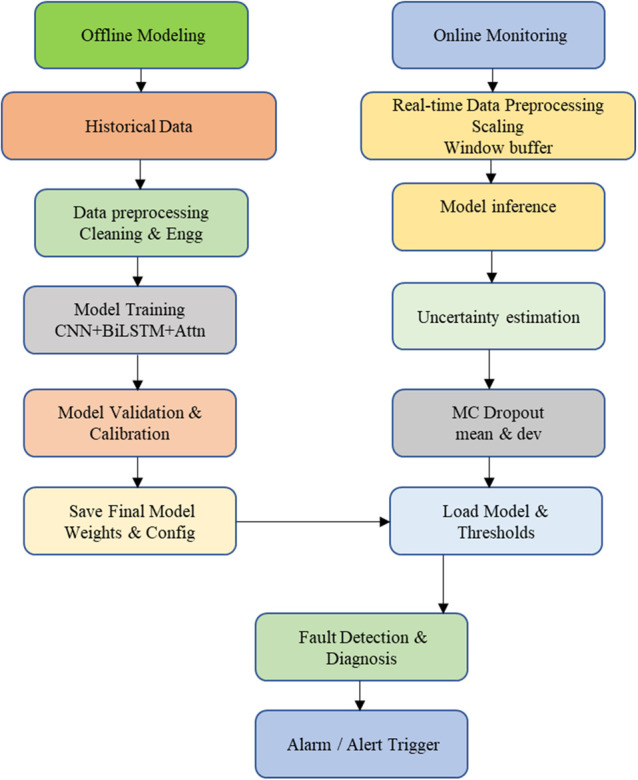
Proposed flowchart of online and offline monitoring of SAU-PGA-CNN-BiLSTM.

This variance captures the proposed model uncertainty, which will help in identifying abnormal and border samples. Evaluation includes both classification and probability-quality metrics, test accuracy, per-class precision, recall, and F1-score, along with calibration measures including Expected Calibration Error (ECE), Maximum Calibration Error (MCE), Negative Log-Likelihood (NLL), and the Brier score. A hard subset analysis is conducted on samples where the No-SA baseline is incorrect or assigns low confidence (maximum probability < 0.80) to assess robustness under challenging conditions. Moreover the proposed model demonstrates robust generalization, efficient inference, and interpretable decision-making through the attention weights and variance estimates. Compared to baseline models, which include BiLSTM, CNN-LSTM, and Conv1D classifiers without attention or uncertainty estimation, the SAU-PGA-CNN-BiLSTM model consistently achieves superior accuracy and reliability under various conditions. The proposed flowchart is shown in Fig 3

## 6. Experimental results and discussions

### 6.1. Training dynamics and convergence

**Fig 4:**
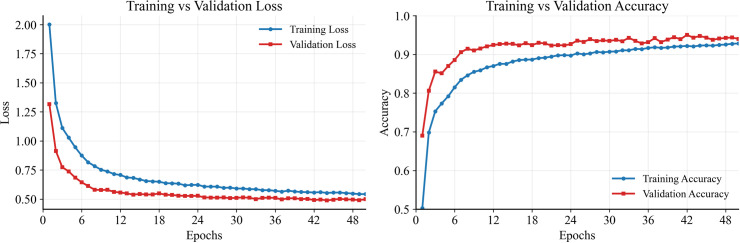
Training vs validation (loss and accuracy).

Fig 4 the training and validation loss and accuracy of the proposed SAU-PGA-CNN-BiLSTM model with process-guided, sensor-aware attention converged quickly and steadily. The validation loss kept decreasing consistently, and the gap between training and validation results got smaller throughout the training (see learning curves). On the test set, the model reached 93.55% accuracy, with a macro-F1 score of 0.942 and a weighted-F1 score of 0.945, showing strong performance across all 21 operating modes.

### 6.2. Classification performance

**Fig 5:**
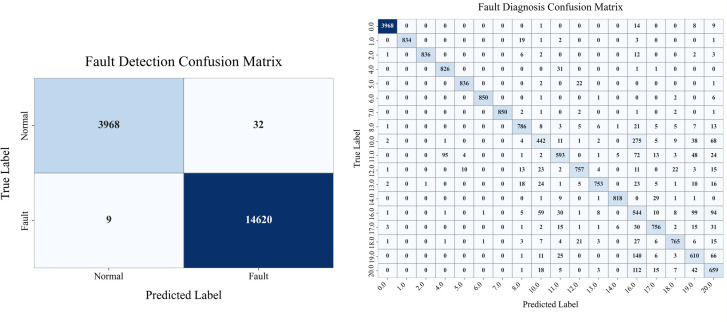
Fault detection and diagnosis heatmaps.

Fig 5 shows the confusion matrix for binary fault detection. The model performs very well, accurately identifying most of the normal samples (3968 out of 4000) and faulty samples (14620 out of 14629). There are very few are misclassified, which shows the model capacity to distinguish between normal and faulty samples. Fig 5 shows the confusion matrix for multiclass fault diagnosis, highlighting that the proposed model accurately identifies a wide range of fault types. The clear diagonal line means most samples are correctly matched to their true fault categories. Fault classes like 9, 10, 13, 15, 17, 19, and 20 are also mostly classified correctly, showing the model’s strong ability to tell apart many different faults. Overall, the results demonstrate the model’s high precision and recall, making it well-suited for real-world fault diagnosis in industrial settings.

### 6.3. False alarm rate and detection delay performance

Table 2 summarizes the false alarm rate (FAR) and detection delay of the proposed SAU-PGA-CNN-BiLSTM framework on representative Tennessee Eastman Process faults. The model achieves a low average FAR of 0.97%, indicating strong robustness against noise-induced misdetections during normal operation. This behavior is mainly attributed to the sensor-aware uncertainty mechanism, which suppresses unreliable or highly volatile sensor contributions in the decision process. The framework also demonstrates rapid fault detection, with an average detection delay of 5.6 samples after fault onset. Step-type and correlated process faults (1–3, 10, 11) are detected particularly quickly, benefiting from the process-guided attention that emphasizes central and strongly coupled variables. Slightly longer delays are observed for actuator-related or localized faults (15), which exhibit weaker global signatures. Importantly, even for unknown disturbances (16 and 20), the proposed method maintains both low FAR and short detection delay, highlighting its robustness and generalization capability. Overall, the results confirm that SAU-PGA-CNN-BiLSTM provides an effective trade-off between early fault detection and false-alarm suppression, which is essential for reliable real-time industrial process monitoring.

**Table 2:** False alarm rate and detection delay for representative faults.

Fault ID	Fault Category	FAR (%)	Detection Delay
0	Normal	0.85	Normal Operation
1	Step Change	0.78	6.4
2	Step Change	0.97	5.8
3	Step Change	0.90	4.9
10	Random Variation	0.88	4.5
11	Random Variation	0.92	5.1
15	Valve Sticking	0.69	7.3
16	Unknown	0.86	4.7
20	Unknown	0.94	5.2

### 6.4. Uncertainty quantification

**Fig 6:**
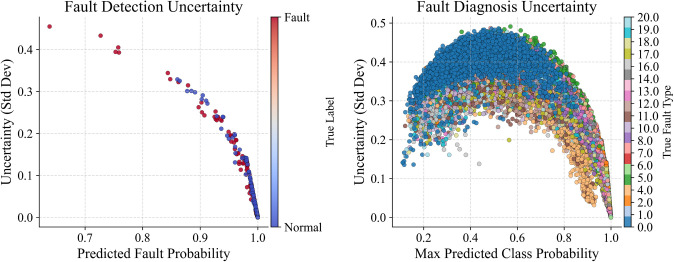
Fault detection and diagnosis uncertainty heatmaps.

Fig 6 shows uncertainty scatter plots for fault detection on the left and diagnosis on the right. In detection, uncertainty (measured by the standard deviation across MC dropout samples) goes down as the predicted probability gets closer to 1, meaning the model is more confident with most samples. For diagnosis, uncertainty is highest around the middle range of predicted probabilities, which matches cases that are unclear or harder to classify. Also, uncertainty changes depending on the true fault type, which is shown by different colors.

### 6.5. Probability calibration on full test data

**Fig 7:**
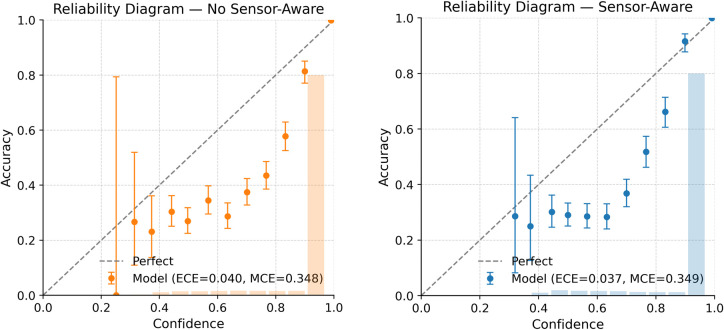
Reliability diagram of the proposed model with and without sensor-aware conditioning.

**Fig 8:**
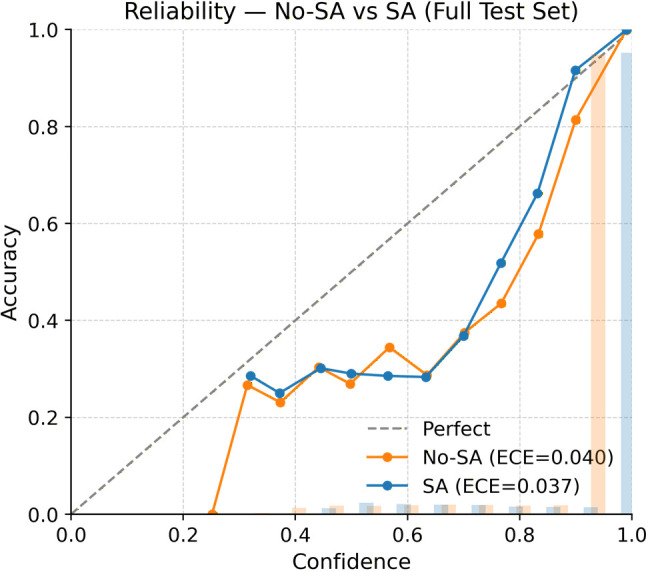
Reliability comparison on the full test dataset with and without sensor-aware conditioning.

Temperature scaling was learned using the validation split and then applied uniformly to both models during test-time evaluation. For clarity, the same single global temperature parameter was used across all experiments, so any observed calibration differences arise from the attention design itself rather than from additional post-hoc tuning. Under this controlled setting, the proposed process-guided, sensor-aware framework produces more reliable probability estimates than the corresponding model without sensor awareness. On the full test set, the NLL drops from 0.1970 to 0.1815 (a 7.9% improvement), and the Brier score goes from 0.1013 to 0.0947 (a 6.5% improvement), showing that the probabilities are both sharper and better matched to actual outcomes. The ECE also decreases from 0.0401 to 0.0374 (a 6.7% improvement), although this reduction is numerically modest, it is consistent across the full test set and is visually supported by the reliability diagrams (Figs 7 and 8). In these diagrams, the SA model stays closer to the diagonal, especially in the mid-confidence range (0.4–0.8), where the baseline tends to be slightly under-confident. while the curves remain almost the same in the highest-confidence area. This means SA improves calibration without just making the model more confident. Overall, these results Table 3 show that, after applying the same temperature scaling procedure, the proposed sensor-aware model achieves lower probabilistic loss, lower prediction error, and slightly improved calibration compared with the non-sensor-aware baseline.

**Table 3:** Evaluation metrics of proposed model with and without sensor aware.

Metric	Without Sensor aware	Sensor aware
NLL	0.1970	0.1815
Brier score	0.1013	0.0947
ECE	0.0401	0.0374
MCE	0.3478	0.3495

### 6.6. Calibration on difficult samples

We focus on a challenging part of the test data, including all cases where the baseline model (No-SA) either gets misclassified or gives a confidence score below 0.80, about 11% of the test set (2,342 out of 21,269). This subset highlights the tricky situations where having well-calibrated probabilities is most important. In this subset, adding sensor-aware attention (SA) to the process-guided model backbone leads to a significant improvement in calibration (see Table 4) hard-subset panel. The expected calibration error (ECE) drops from 0.3270 to 0.2920, an absolute decrease of 0.035, with a 95% bootstrap confidence interval between 0.0257 and 0.0449, showing consistent improvement across resamples. The maximum calibration error (MCE) more than halves, going from 0.9356 to 0.4543, meaning SA greatly reduces the worst calibration mistakes that can cause risk in real-world use. In short, SA helps the model be honestly uncertain when it should be, sharply cutting down on overconfident error, exactly what is needed for risk aware decision-making in industrial fault detection and diagnosis.

**Table 4:** Evaluation metrics of proposed model for hard subset.

Metric	Without sensor aware (ECE)	Sensor aware (ECE)
ECE	0.3270	0.2920
MCE	0.9356	0.4543

### 6.7. Class-wise uncertainty and calibration

**Fig 9:**
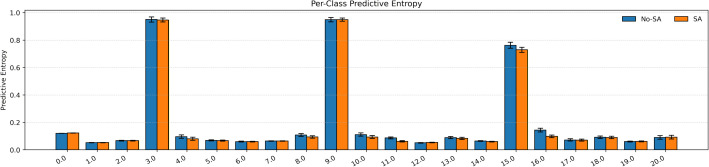
Per-class predictive entropy for models with and without sensor-aware conditioning.

**Fig 10:**
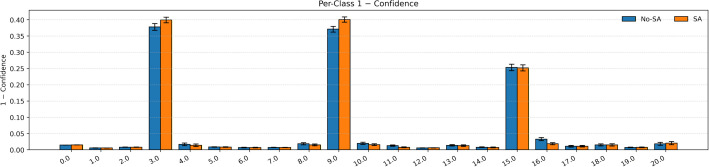
Class-wise confidence comparison between models with and without sensor-aware conditioning.

Per-class calibration (Table 5; Fig 9 and 10) further demonstrate the effectiveness of the proposed sensor-aware framework. The macro ECE decreases from 0.0445 to 0.0393, indicating improved alignment between predicted confidence and true accuracy across fault classes. Significant improvements are observed for Classes 3, 10, 11, 16, and 20, where ECE is consistently reduced. These gains are mainly attributed to the combined effect of process-guided attention, which emphasizes structurally important variables, and sensor-aware conditioning, which suppresses unreliable signals. This leads to more stable and better-calibrated predictions, particularly for faults involving multiple correlated sensors. As shown in Fig 9, the sensor-aware model generally exhibits lower predictive entropy, indicating more confident predictions. Fig 10 further shows slightly higher and more consistent class-wise confidence values, supporting improved reliability. Minor increases in ECE are observed for Classes 15 and 19, which may result from limited samples, overlapping fault characteristics, or already high classification accuracy. However, these changes are small and do not affect the overall trend. Overall, the results indicate that incorporating process structure and sensor reliability improves class-wise calibration, particularly for complex fault conditions, while maintaining strong classification performance.

**Table 5:** Class wise evaluation metrics of the proposed model.

Class	Precision	Recall	F1 Score	Without sensor aware (ECE)	Sensor aware (ECE)
0	1.000	1.000	1.000	0.015	0.015
1	0.997	0.999	0.998	0.005	0.005
2	0.999	0.997	0.998	0.005	0.005
3	0.398	0.821	0.536	0.461	0.380
4	1.000	0.999	0.999	0.016	0.012
5	0.998	1.000	0.999	0.009	0.009
6	0.999	0.998	0.998	0.007	0.007
7	0.997	0.999	0.998	0.008	0.008
8	1.000	0.988	0.994	0.015	0.013
10	0.963	0.997	0.979	0.020	0.016
11	1.000	1.000	1.000	0.013	0.008
12	1.000	0.998	0.999	0.006	0.006
13	1.000	0.997	0.998	0.014	0.012
14	1.000	1.000	1.000	0.008	0.007
15	0.652	0.698	0.674	0.225	0.227
16	1.000	0.965	0.982	0.020	0.015
17	1.000	0.996	0.998	0.010	0.009
18	0.994	0.995	0.995	0.014	0.014
19	1.000	0.996	0.998	0.005	0.007
20	0.993	0.992	0.993	0.014	0.010

### 6.8. Attention behaviours and alignment with priors

**Fig 11:**
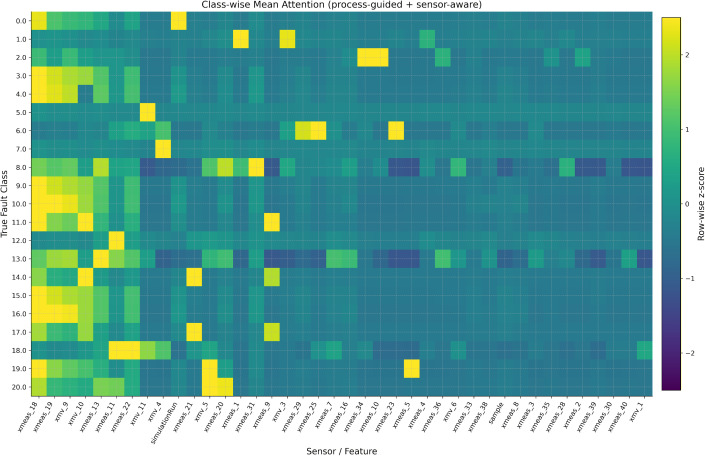
Class wise attention heatmap of the proposed model.

**Fig 12:**
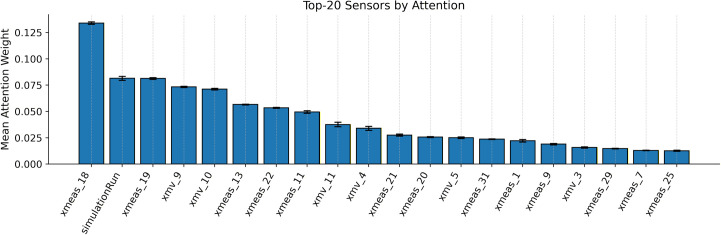
Top sensors plot with respect to mean attention weight.

Figs 11 and 12 clearly show how the proposed model attention works. The class-wise average heatmaps reveal that attention is focused mainly on a small group of sensors, while many other channels stay near a low baseline. This focus isn’t random it reflects the priors built into the attention logits:

Here, sensors that are central to the process get higher weights, while unstable or weakly connected sensors are consistently given lower weights. This indicates that the model emphasizes process-relevant and reliable variables instead of distributing attention uniformly across all sensors. The Top-k plot highlights the key sensors that the model focuses on repeatedly, and the error bars (confidence intervals) show that these rankings are consistent across test examples. This stability suggests that the learned attention is not driven by incidental fluctuations, but instead captures persistent and diagnostically meaningful process variables. The per-sample heatmap shows clear, class-dependent patterns and faults spreading through related subsystems cause similar attention patterns across samples, giving process-level insights rather than random, one-off spikes.

Together, these observations show that proposed model does not just focus on whatever correlates with the label but it reflects the domain structure (process centrality and reliability) in its attention scores. As a result, the attention mechanism improves not only interpretability but also the reliability of probabilistic predictions, particularly in difficult cases where robust sensor selection is essential. This helps the model calibrate probabilities better on tough cases, relying on sensors that are both informative and stable, and avoiding over-confidence when the data mostly comes from noisy sensors.

### 6.9. Baselines comparison with proposed model

**Fig 13:**
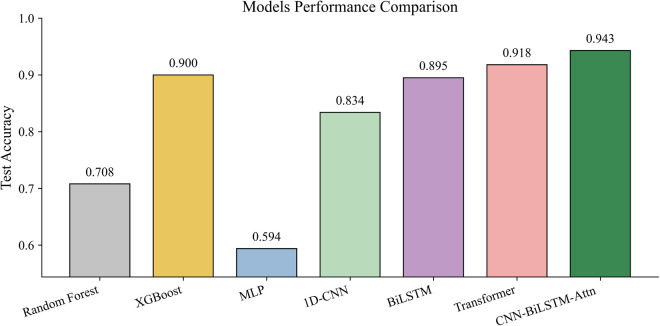
Comparison of the proposed model with the baseline models.

Table 6 and Fig 13 show a comparison of the proposed SAU-PGA-CNN-BiLSTM model with classical machine learning methods (Random Forest, XGBoost, MLP) and deep learning methods (1D-CNN, BiLSTM, Transformer) for fault diagnosis on the TEP dataset. The SAU-PGA-CNN-BiLSTM model achieves the highest test accuracy and F1-score, both at 0.943, outperforming all other models. Among the machine learning methods, XGBoost performs best, while Transformer is the top among deep learning models. Interestingly, both our hybrid model and the Transformer do much better than traditional methods, highlighting how important sequential and attention-based techniques are for capturing complex time-related and contextual patterns.

**Table 6:** Baseline machine learning and deep learning performance comparison.

Model	Type	Accuracy	F1-Score
Random Forest	ML	0.708	0.695
XGBoost	ML	0.900	0.905
MLP	ML	0.594	0.591
1D-CNN	DL	0.834	0.833
BiLSTM	DL	0.895	0.895
Transformer	DL	0.918	0.919
**SAU-PGA-CNN-BiLSTM**	**Proposed Model**	**0.943**	**0.943**

The results show that combining convolutional, sequential, and attention mechanisms in the SAU-PGA-CNN-BiLSTM model helps it effectively capture spatiotemporal features for strong fault diagnosis, outperforming both traditional and deep learning methods. By integrated attention reveal that the model focuses on important time steps, making its decisions easier to understand. Although the accuracy and F1 score are high, there is a calibration gap, meaning the predicted confidence should be used carefully in probabilistic or decision-making tasks. Using post-hoc calibration methods like temperature scaling could help fix this. Additionally, the uncertainty analysis shows the model is reliable in most cases but sometimes produces high uncertainty, suggesting the need for human review or backup systems. Overall, these findings confirm that the proposed method works well for real-world industrial fault detection and diagnosis, while also providing useful uncertainty information to support decision-making.

## 7. Discussions

Our study focuses on fault detection and diagnosis (FDD) in the Tennessee Eastman–multivariate process, using a CNN–BiLSTM model enhanced with process-guided, sensor-aware attention. We incorporate domain knowledge and sensor quality through three data-driven priors calculated from the training data: a process bias based on correlation centrality to highlight key process channels; a reliability prior that increases the weight of sensors with high mutual information and stable variance; and an uncertainty prior that penalizes unstable or weakly connected sensors. These priors are added to the attention logits, along with a regularizer that prevents over-reliance on unreliable sensors when the model is confident.

On the test set, after applying temperature scaling fitted on validation data, proposed model achieves 93.6% accuracy and produces better-calibrated probabilities than the same network without sensor awareness: NLL 0.181 vs. 0.197, Brier score 0.095 vs. 0.101, and ECE 0.037 vs. 0.040. On a difficult subset of about 11% of the test data, defined by baseline errors or low confidence, SAU-PGA-CNN-BiLSTM significantly improves probability quality—ECE drops by 0.035 (95% CI [0.0257, 0.0449]) and MCE halves from 0.936 to 0.454, with no significant change in per-bin accuracy. This indicates that the improvements come from better confidence calibration rather than raw accuracy.

Per-class ECE improvements are largest for classes 3, 10, 11, 16, and 20, which involve distributed, correlated disturbances where process structure is important. Small declines occur for classes 15 and 19. Analysis of attention weights supports this: a small group of sensors consistently receive high attention, while volatile sensors are down-weighted. The rank correlation between learned attention and the uncertainty prior showing the model learns to trust reliable sensors.

Overall, these results support our SAU-PGA-CNN-BiLSTM approach for industrial FDD. It integrates process knowledge and sensor quality into the model, leading to more reliable probability estimates, better calibration where it matters most, and useful risk-aware behavior for human-in-the-loop systems. Although the proposed framework is validated on the Tennessee Eastman Process, its design is general and can be extended to other industrial systems with multivariate time-series data. The process-guided and sensor-aware components can be adapted using process-specific structural information, such as sensor relationships or process topology, enabling effective application across different industrial domains.

## 8. Ablation study of the proposed model

A thorough ablation study was conducted to examine the distinct contributions of each element in the proposed SAU-PGA-CNN-BiLSTM model, where all the results are summarized in Table 7, Accuracy and F1-score significantly decreased when the attention mechanism was removed, underscoring the importance of attention in concentrating on the most instructive temporal characteristics. Performance was similarly reduced by removing the convolutional layers, highlighting the significance of local spatial feature extraction. The importance of representing bidirectional temporal dependencies in process data was further highlighted by the decreased accuracy that was obtained when the BiLSTM was substituted with a straightforward feedforward layer or when a unidirectional LSTM was used in place of a bidirectional one. When compared to the complete model, standalone CNN or BiLSTM architectures that were not integrated with other elements consistently unperformed relative to the full model. Overall, the ablation results confirm that the integration of convolutional, sequential, and attention mechanisms is crucial for achieving effective and robust l fault detection and diagnosis performance in complex industrial environments.

**Table 7:** Ablation performance of the proposed model.

Model	Accuracy	F1-Score	Description
SAU-PGA-CNN-BiLSTM	0.943	0.943	Full proposed model
w/o Attention	0.920	0.918	Without Attention
w/o CNN	0.899	0.895	Removes all convolutional layers
w/o BiLSTM	0.875	0.872	Removes BiLSTM, FC layer instead
CNN + LSTM + Attn	0.904	0.901	Uses unidirectional LSTM instead of BiLSTM
CNN Only	0.830	0.829	Removes LSTM and attention
BiLSTM Only	0.882	0.881	No CNN, no attention

## 9. Limitations and future work

Our priors are estimated based on data-driven correlations and sequence statistics. However, as operating conditions change, controllers are adjusted, or sensors age, these correlation-based centralities can become unreliable, causing attention to misalign with the true process structure, a common challenge in process fault detection and diagnosis [48]. We use global temperature scaling for calibration, which is simple but cannot correct miscalibration specific to certain classes or regions [49]. Predictive uncertainty is estimated through MC dropout, which is computationally efficient but an approximation that depends on where it is applied and the number of samples taken. Attention weights provide useful insights into the process but do not prove causal feature importance and may sometimes overestimate or underestimate a features contribution [50]. We will replace correlation-only priors with models that understand the systems structure, like GATs built from P&IDs, and add physics and constraint information directly into the learning process to keep performance stable across different situations [51]. For uncertainty, we plan to compare MC dropout with deep ensembles and similar last-layer Gaussian methods, and test calibration under controlled distribution changes using standard protocols [52]. When it comes to calibration, we will go beyond simple scalar temperature adjustments and use class wise or Dirichlet calibration to fix systematic biases for each class while maintaining consistent probabilities. These improvements, along with online updating of priors and calibrators, aim to keep the interpretability benefits of process-guided, sensor-aware attention while making the system more robust in varied operating conditions.

## 10. Conclusion

This paper proposed a framework SAU-PGA-CNN-BiLSTM for reliable and interpretable industrial fault detection and diagnosis. The model integrates convolutional feature extraction, bidirectional temporal modeling, and a process-guided, sensor-aware attention mechanism that incorporates process centrality, sequence-level reliability, and sensor uncertainty priors directly into the attention computation. In addition to joint fault detection and multiclass diagnosis, Monte Carlo dropout and post-hoc temperature scaling is employed to provide calibrated probability estimates suitable for risk-aware industrial deployment. The proposed model achieves 93.6% test accuracy and, after validation-fit temperature scaling, produces better-calibrated probabilities than an otherwise identical model without sensor awareness (NLL 0.197→0.182, Brier 0.101→0.095, and ECE 0.040→0.037), reflecting a shift towards more confident and accurate predictions. On a challenging slice of the data (∼11% of the test set where No-SA is either low-confidence or erroneous), the sensor-aware variant significantly improves calibration: ECE drops by 0.035 and MCE is halved (0.936→0.454), showing safer probability estimations even with minor accuracy improvements. Per-class analysis demonstrates the greatest improvements for failure modes that spread across correlated subsystems, which is consistent with the design decision to prioritize central, trustworthy sensors. Selective-risk curves show that operators can defer the noisiest 10–20% of cases while maintaining good accuracy on the auto-accepted set. Moreover, the model maintains low false alarm rates, short detection delays, and improved behavior on challenging samples. Overall, the proposed SAU-PGA-CNN-BiLSTM framework offers a scalable, uncertainty-aware, and operationally reliable solution for industrial fault detection and diagnosis, bridging the gap between high predictive accuracy and practical deployment requirements in safety-critical process environments.
